# Molecular Melodies: Unraveling the Hidden Harmonies of NMR Spectroscopy

**DOI:** 10.3390/molecules29040762

**Published:** 2024-02-07

**Authors:** Iria Pérez Varela, Gavin Shear, Carlos Cobas

**Affiliations:** 1 Centro de Investigación Mestrelab (CIM), Av. Barcelona 7, 15706 Santiago de Compostela, Spain; iria.perez@mestrelab.com; 2 Mestrelab Research, 15706 Santiago de Compostela, Spain; gavin.shear@mestrelab.com

**Keywords:** NMR, FID, sound

## Abstract

This work explores the evolution of auditory analysis in NMR spectroscopy, tracing its journey from a supplementary tool to visual methods such as oscilloscopes, to a technique sidelined due to technological advancements. Despite its renaissance in the late 1990s with artistic and scientific applications, widespread adoption was hindered by the necessity for hardware modifications and reliance on specialized software. Addressing these barriers, this paper introduces a new feature in Mnova NMR software that facilitates the easy auditory interpretation of NMR signals. We discuss new applications of this tool, emphasizing its utility in aiding the identification of specific functional groups by auditory analysis of the spectrum’s multiplets, such as distinguishing between aromatic, olefinic, or aliphatic protons, thereby enriching the interpretative capabilities of NMR data.

## 1. Introduction

Nuclear magnetic resonance (NMR) has long been a cornerstone technique in various scientific fields, ranging from chemistry to medicine, and from the in-depth characterization of materials to the intricate analysis of molecular interactions such as protein-ligand bindings. Its unparalleled ability to provide insight into molecular structures, dynamics, and physical properties has made it an indispensable tool in both fundamental and applied research domains. Over the decades, as the technology behind NMR has evolved, so too have the ways in which scientists engage with and interpret the data it produces. One lesser-known yet captivating dimension of this history is the auditory exploration of NMR signals.

In the nascent stages of NMR spectroscopy, auditory monitoring of NMR signals was a complementary tool, enhancing the traditional visual methods like oscilloscopes to facilitate signal analysis. Pioneers like Felix Bloch and Edward Purcell, who independently crafted the early NMR methodologies, typically channeled the detected free induction decay (FID) signals through amplifiers and into loudspeakers [1,2]. This was not merely an idiosyncratic method; it offered real-time feedback, enabling scientists to ascertain resonances without the continuous scrutiny of oscilloscopes or similar devices. As the field evolved, this auditory approach was not just confined to the continuous wave (CW) era. By the late 1960s, with the advent of Fourier transform (FT) NMR, such practices became more pronounced. Scientists could discern a well-tuned NMR probe from a poorly tuned one through clear auditory cues. The sounds—ranging from distinct “swooshes” to faint or distorted noises—proved helpful for tasks like tuning the probe. For instance, a shorter sound often indicated poor shimming and field inhomogeneity, while a longer, sustained sound suggested better magnetic field homogeneity achieved through optimized shimming.

However, as NMR technology advanced and digital data processing became more prevalent, the practice of acoustically listening to the NMR signal diminished. By the late 20th century, especially in the 1980s and 1990s, with the increasing sophistication of computer interfaces and the dominance of digital processing, the auditory dimension of NMR became somewhat archaic.

Although the allure of sound within the scientific realm of NMR had faded, Walter Bauer of the University of Erlangen–Nürnberg reintroduced and expanded upon this auditory dimension [3]. Playfully capturing this union of sound and spectroscopy, he coined the acronym NMR Meets MUSICIANS (multifrequent utility for sound-inspired crazy and insane abuse of new spectrometers).

Bauer’s approach was an intriguing mix of the old and new. He devised a method to generate audio by directly interfacing with the NMR spectrometer’s circuitry, tapping into it before the audio-to-digital converter. This allowed him to capture the analogue FID signal in its pure form before any digitization occurred. It is essential to note that this FID was in the audio frequency range, available in the receiver path due to the down-conversion which is a standard step in any transient NMR signal detection (see later). His choice of acetone for 1H NMR spectra was strategic due to its single resonance. Bauer retrofitted an NMR JEOL instrument to capture the pure sound of acetone. By manipulating the carrier frequency in the experiment’s pulse program file and adjusting time delays, he was able to generate distinct sounds, akin to different notes, which could be woven together into melodies, including classical legends like Bach and the Beatles.

Walter Bauer’s innovative approaches delved into an interplay between the science of listening to NMR spins in their inherent form and the art of melody composition. This synthesis of raw NMR auditory experiences and melodic reinterpretation pioneered a movement where molecular spins began to be experienced as melodies.

Expanding on this foundation, various researchers ventured further, crafting intricate musical compositions from 1H [4] and 13C NMR [5], and even 2D NMR spectra [6]. While this approach offered a richer auditory landscape, it often prioritized artistic interpretation over the direct portrayal of natural spin precessions. For a more detailed exploration of this intersection of NMR and art, see reference [4]. Notably, the author introduces the concept of *Molechord*. Drawing parallels to music, if a molecule producing a single resonance can be likened to a musical pitch, then a molecule with multiple resonances can be conceptualized as a chord. This concept of translating spectroscopic data into audible forms is not exclusive to NMR. Similar endeavors have been undertaken in other spectroscopic techniques, such as infrared spectroscopy [7].

All the applications discussed earlier required either direct intervention in the spectrometer’s hardware or specialized software tools to convert the digital NMR data into audio files (e.g., WAV files) for playback. These complexities have deterred many NMR users from experiencing the auditory sensation of NMR signals, given the lack of a straightforward method to achieve it.

To bridge this gap and offer this capability to the scientific community, we have introduced a new feature in Mnova NMR [8] that enables users to audibly render NMR signals with a simple click of a button. Our goal has been to ensure this playback is as faithful as possible to the actual natural spin frequencies and can be heard clearly once the original signals in the MHz range have been converted in the spectrometer into the audio frequency range. While this might not be a strict representation of the raw physical process, in this context, it is the closest approximation to reality we can achieve. Moreover, since the most widespread acquisition method employs quadrature detection [9,10], it is necessary to process the FID in a special way to ensure that the frequencies heard in the audio match the observed nuclides frequencies in the spectrum. This operation, as well as some practical applications, will be discussed next in this article.

In this work, we also introduce a novel approach that goes beyond the traditional acoustic reproduction of the FID. This method is tailored to allow listeners to focus on specific regions of interest within the spectrum, presenting a potential, albeit rudimentary, tool for molecular structure elucidation.

Eight 1H NMR spectra and sixteen sound files are provided in the Supplementary Materials, representing the two distinct playback modes delineated in this work

## 2. Discussion

### 2.1. Introduction to the Physical Origins of NMR Resonances

NMR is a multifaceted technique encompassing spectroscopy, imaging, and relaxometry. Its foundational concept lies in the nuclear spin, an intrinsic property of certain atomic nuclides. When exposed to an external magnetic field, these spins, each possessing a unique magnetic moment, tend to align themselves along the field (polarization). However, this alignment is countered by the random thermal fluctuations of the molecules, which drive the system towards a state of random magnetic moment orientations. The randomizing effect of thermal motion is not complete, leaving a small energetic advantage for the magnetic moments to align with the field. Consequently, the magnetic moments of all nuclides in a sample sum up to a macroscopic vector known as nuclear magnetization. In equilibrium, nuclear magnetization aligns along the magnetic field, but being tiny and static, it is nearly impossible to detect against the main external field. Applying a suitable radiofrequency pulse can rotate the nuclear magnetization to any desired angle, bringing it into a non-equilibrium state where it is no longer aligned with the field, a process termed excitation. The excited nuclear magnetization component, which is transversal to the magnetic field, precesses around the field direction with a frequency proportional to the external field. This is known as the Larmor frequency, typically in the radiofrequency (RF) range.

The excitation pulse must have a carrier frequency close to the Larmor frequency (the resonance condition) to be effective, thus typically exciting specific nuclides like 1H or 13C, but not both simultaneously. The precessing nuclear magnetization component, although small, is detectable, as it induces RF signals in a nearby receiver coil, leading to the concept of nuclear induction. After excitation, nuclear magnetization returns to its equilibrium state, a process known as relaxation. During this relaxation phase, the receiver coil detects the transient signal, referred to as free induction decay (FID).

If all nuclides in a sample perceived the same magnetic field, the scope of NMR would be markedly narrow. However, the synergy of the following factors significantly enriches the capabilities and applications of NMR, transforming it into an exceptionally powerful technique:

(1) Nuclides are partially shielded from the external magnetic field by the electron cloud surrounding them, which modifies the local magnetic environment. These variations, known as chemical shifts, form the cornerstone of NMR spectroscopy, enabling its vast applications in chemistry. A nucleus within an atom or molecule feels a slightly different field than a bare nucleus due to the external field prompting electron circulation within atomic orbitals. This induced electron motion generates a small counteractive magnetic field. The resonance frequency of a nucleus is distinctive of its specific chemical environment. Greater electron density around a nucleus translates to greater shielding, resulting in a lower NMR frequency compared to the same nuclide in a less shielded environment. Furthermore, the anisotropy of electron distribution in certain molecular structures, such as aromatic rings, introduces additional considerations. In aromatic compounds, the delocalized π electrons form a circulating ring current when exposed to a magnetic field. These currents generate their own magnetic field, affecting nearby nuclides differently based on their spatial relation to the ring. Nuclides in the plane of the ring experience reduced shielding, while those perpendicular to the ring might be shielded, producing unique chemical shifts.
(2) Local fields vary at a nuclide’s site due to their interactions with neighboring nuclides. These may be either direct (dipolar; the fields of close-by magnetic dipoles) or indirect (scalar; mediated by chemical bond electrons). In the case of 1H NMR spectra, the latter are responsible for the well-known multiplet patterns.
(3) Intentionally produced field variations, achieved through precisely controlled magnetic field gradients, modulate the magnetic field in each voxel of the sample according to its spatial location. This principle underpins the technical foundation of magnetic resonance imaging (MRI).

The detected NMR signal (FID or interferogram) is therefore a linear superposition of decaying harmonics produced by all the chemically distinct nuclides in the sample. While various mathematical procedures exist to analyze this NMR signal, the Fourier transform stands out as the most commonly employed technique. This method meticulously deconstructs the signal into its constituent frequencies. Mirroring this mathematical process through a corresponding natural mechanism, our ears systematically perform a similar operation. Within the cochlea reside hair cells, each tuned to resonate at a specific frequency. As sound waves enter the ear, these cells individually isolate and identify the different frequency components, akin to a biological version of the Fourier transform.

### 2.2. Audification Methodology

Mnova has steadily established itself as one of the most widely used software tools for NMR data processing, analysis, and prediction. A standout feature of Mnova is its capability to import NMR data from an extensive array of instruments, including both high-field and benchtop spectrometers. This broad compatibility paves the way for a universal method of acoustic rendering of NMR spectra, overcoming the limitations posed by brand-specific data formats and ensuring a cohesive user experience. Furthermore, its multiplatform compatibility (Win/Mac/Linux) ensures that researchers from diverse computational backgrounds can benefit equally.

The new functionality in Mnova NMR allows users to listen to both the original FID as it comes from the instrument and the processed FID. The processed FID includes the data that has undergone time–domain operations such as apodization and zero-filling, amongst others, that is, those applied right before the Fourier transform. This feature not only offers deeper insights for users but also serves as a valuable educational tool. For example, with ^13^C spectra that have a low SNR, the initial audio may be noisy. After an exponential apodization function is applied, the sound changes, enabling users to discern the difference and understand the effects of the preprocessing. 8 ^1^H NMR spectra and 16 sound files are provided in the Supplementary Materials. Reference [8] is cited in the Supplementary Materials.

### 2.3. NMR to Audio: Resolving Quadrature and Aliasing

In this work, we aim to ensure that the acoustic reproduction of the NMR signal accurately reflects the frequencies identified in the spectrum. Notably, due to the complex (in the mathematical sense) nature of the NMR signal and the inherently real nature of sound, the pitch heard may not necessarily align with the resonances observed in the NMR spectrum. This discrepancy can arise from various acquisition parameters, including the spectral width. In this section we will introduce the signal processing formalism essential for harmonizing the frequencies represented in the NMR spectra with those experienced in the auditory output.

In NMR spectroscopy, the intrinsic Larmor frequencies of signals lie in the MHz range. Detecting these high frequencies directly poses challenges due to sensitivity constraints and electronic limitations. To address this, NMR signals undergo a down-conversion to a more manageable frequency through “heterodyning”. This technique mixes the original NMR signal with a reference frequency, producing an intermediate frequency (typically between 10 kHz and 100 kHz), which is then amplified and filtered to fit within the audio range.

Quadrature detection, now universally available on all modern NMR spectrometers, utilizes two phase-sensitive detectors with a 90° phase difference and strategically positions the carrier frequency at the spectrum’s center. This configuration results in signals with positive or negative frequencies, reflecting their frequencies relative to the carrier, reducing spectral width and folding of noise. In this scheme, the RF signal from one channel is treated mathematically as the real signal while the other as the imaginary signal. When combined in a complex Fourier transform, positive frequency signals are differentiated from negative frequency signals, overcoming the limitations of single-channel detection.

By convention, NMR spectra are plotted with the frequency increasing from right to left, with the most shielded nuclides (those with lowest chemical shift) on the right. To achieve this, the output of the Fourier transform needs to be rotated by half the spectral width (i.e., 1/(2Δ*t*) or N/2 data points in the frequency domain) so the NMR spectrum covers the range −1/(2Δ*t*) to (N/2 − 1)/(NΔ*t*) [11].

While quadrature detection effectively differentiates between positive and negative frequency signals in the NMR spectrum, this acquisition scheme introduces challenges when converting the FID signal into an audible format. The principal issue stems from the inherent discrepancy between NMR spectroscopy and audio processing: NMR employs both real and imaginary components to discern positive and negative frequencies, whereas audio reproduction is based solely on the real component. Consequently, omitting the imaginary part during audio conversion leads to aliasing, where high-frequency signals surpassing the Nyquist frequency (half the spectral width) are reflected back into the lower half of the spectrum. This reflection results in the superimposition of high-frequency components onto lower frequencies, thereby distorting the audio representation and leading to potential misinterpretation of the audio output.

**Figure 1:**
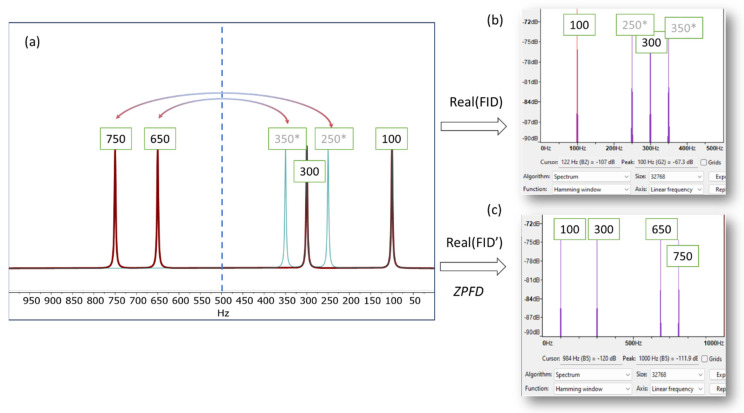
(**a**) Simulated spectrum displaying resonances at 100, 300, 650, and 750 Hz (indicated by the red line), with a spectral width (*SW*) of 1000 Hz. When using only the real part of the FID to generate the audio file, frequencies exceeding half of the *SW* (i.e., above 500 Hz) will fold back into the right half of the spectrum (green lines, marked with an asterisk (*) in the figure). This phenomenon is depicted in (**b**), where the spectrum plot from Audacity is employed to demonstrate the frequencies that would be perceptible in the audio. (**c**) Shows the Audacity plot generated using the augmented FID, following the zero-padding frequency domain (ZPFD) procedure described in the text. The frequencies audible in this plot correspond accurately to those in the NMR spectrum.

This is illustrated in Figure 1, which presents a simulated NMR spectrum with a spectral width of 1000 Hz, containing four signals at 100, 300, 650, and 750 Hz. To provide a basic validation, we use the software program Audacity (Version 3.4.2) [12] and its analysis tool, which plots the Fourier transform of the audio generated from the NMR signal. When only the real part of the FID is used, the resulting audio file exhibits aliasing frequencies, specifically at 250 and 350 Hz in this instance. As a result, the higher frequencies are not accurately conveyed in the audio, leading to an auditory experience that does not accurately reflect the nuanced details of the original NMR signal.

**Figure 2:**
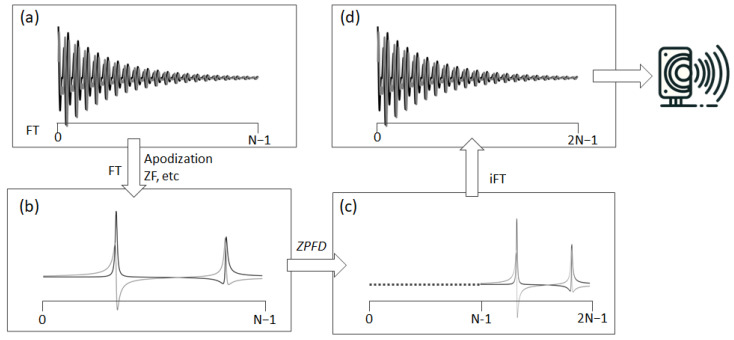
This figure depicts the transformation of an NMR signal from its acquisition to its preparation for auditory representation. It begins with (**a**) the original complex FID, acquired in quadrature. The process then advances to (**b**) the processed frequency domain spectrum, which is obtained from standard time domain operations and FT. Following this, (**c**) ZPFD is applied, doubling the number of data points in the spectrum by adding zeros to the left side. This step leads to (**d**) the inverse FT, resulting in the augmented FID with a doubled sampling rate. This augmented FID, with its increased number of data points, is now ready for conversion into an audio file.

To effectively resolve aliasing and fully leverage the acquired spectral information, we have devised a special signal processing technique we term ZPFD (zero padding in the frequency domain). This approach incorporates the entire spectral data, including the imaginary part that was initially disregarded during the audio file generation. The goal is to transfer the imaginary component into the real part, thereby creating an augmented real component that encapsulates the entire spectral information and therefore eliminating the aliasing artefacts. This processing scheme consists of the following steps (Figure 2):

(1) Starting with the original FID, it is processed using standard techniques to obtain the frequency domain spectrum (FD). During this phase, functions such as apodization, zero-filling, or any other necessary operations can be applied.
(2) Once in the FD, if the spectrum contains N complex points, N zeros are added through zero padding at the beginning, i.e., to the left of the complex vector representing the digital points of the spectrum. This zero padding effectively doubles the number of points in the FD spectrum.
(3) Finally, an inverse Fourier transform (FT) is performed on this expanded dataset, now comprising 2N points. The sampling rate in the audio file is then set to twice the original spectral width. The real part of this augmented FID is ready to be converted into an audio file.

The fundamental principle of this technique involves expanding the FD spectrum through zero padding. This augmentation translates into an interpolation of the new FID after FT of the expanded spectrum. Such interpolation effectively inserts a new point between each pair of existing data points, thereby doubling the rate of digitization. An alternative method to achieve a similar outcome involves padding N/2 zeros both to the left and right sides of the spectrum. This method, however, requires an additional step: the adjustment of all signals by shifting them by half the spectral width, repositioning the carrier frequency to one end of the spectrum. Owing to its simplicity and efficiency, the first method—zero padding on one side—is preferred and adopted in this study.

In NMR spectroscopy, it is customary to reference the frequency scale using a reference signal, such as TMS. As a result, the rightmost frequency in the NMR spectrum does not correspond to 0 Hz. Instead, it takes on an offset determined by the reference standard. This referencing leads to all spectral frequencies being shifted by a constant value. As a consequence, for the acoustic rendition of the FID to accurately mirror the visually observed frequencies in the spectrum, this frequency offset needs to be accounted for. Shifting the frequencies in the time domain can be efficiently performed by multiplying the FID by a complex exponential function:

$$e^{-i2\pi ∆ft}$$

This ensures that the frequency referencing observed visually in the NMR spectrum aligns correctly with the acoustic representation.

## 3. Applications

### 3.1. Educational Echoes: NMR in the Classroom

One of the most promising realms for the application of auditory NMR is education. Traditional pedagogy has relied heavily on visual aids, but auditory tools can bring forth a unique perspective that caters especially to visual or auditory learners. The study by Munukutla et al. exemplifies this [13]. In their work, they transformed NMR spectra into sound, giving students a novel way to interact with and understand NMR data.

However, the study also highlighted the need for a more streamlined approach. The method Munukutla et al. employed required various steps, including manipulating data in Excel and using third-party software like Garageband or Logic Pro. Additionally, for real-time playback, specific hardware setups were required. This multistep, multi-software approach can be cumbersome, especially for beginners. The hope is that the new auditory NMR tool implemented in Mnova will overcome these limitations by offering users a more direct and seamless experience, allowing them to easily listen to their spectra without the intricate setup.

Beyond just listening, this tool has potential applications in explaining NMR signal processing concepts. Consider the principle of apodization. This process, used to improve the signal-to-noise ratio (SNR) in NMR (amongst other applications like resolution enhancement), can be hard for students to conceptualize. With the auditory NMR tool, students can literally hear the changes as different apodization parameters are adjusted. The shifts in SNR and the decay rate become audibly apparent. Noise levels change, and the speed of decay—directly proportional to the spectra linewidths—can be discerned. Such a hands-on (or perhaps ears-on) approach fosters an intuitive understanding that traditional methods might not achieve.

#### Audible Indicators for Instrument Calibration and Control

The sound derived from NMR data, while not necessarily the most efficient method for all users, can act as an immediate indicator of the instrument’s calibration status. Distinct auditory patterns might swiftly alert an operator if the device is adequately tuned or shimmed. While the practical applications of this method might lean more towards educational purposes than everyday real-world utility, its conceptual significance should not be overlooked.

Equally, in sequential or arrayed NMR experiments such as reaction monitoring or relaxation studies, auditory analysis of the FID can be beneficial. This approach allows for real-time auditory confirmation of various experimental parameters, such as the efficacy of water suppression. Immediate corrective actions can be taken if deviations are detected. Additionally, this auditory method could potentially facilitate the identification of significant changes in signal intensity, such as the relative decrease of a reactant signal compared to a product, thereby providing an innovative and interactive way to monitor reaction progress.

By integrating the auditory dimension in NMR spectroscopy, we highlight the potential for broader accessibility and the chance to explore more diverse research methodologies, even if some of them might be more conceptual than practical in nature.

### 3.2. Incorporating Sound for Enhanced Accessibility

Integrating auditory elements into scientific research can offer unique advantages. In the specific context of NMR spectroscopy, the auditory representation of data can contribute to both inclusivity and instrument efficiency.

#### A Tool for Educational Support and Enhanced Accessibility

NMR spectroscopy, like many scientific disciplines, predominantly relies on visual data representation. While an auditory rendition of NMR spectra might not substitute the comprehensive visual analysis that researchers typically depend on, it can serve as a supplementary tool. For those with visual impairments, listening to NMR data can offer an added layer of information. This becomes especially beneficial in educational contexts, where auditory cues can assist in explaining basic concepts or phenomena (vide supra). Moreover, for quick checks or fundamental interpretations of data, the auditory component might offer a more intuitive grasp.

In this work, we introduce a novel approach that can serve, for instance, as a rudimentary structure elucidation tool, especially beneficial for visually impaired users. This unique feature emphasizes specific regions of the spectrum, allowing listeners to acoustically discern, for example, distinct functional groups or specific types of protons. The details of this method are elaborated in the subsequent section.

### 3.3. Acoustic Mapping of Spectral Regions for Novel Structure Interpretation

In the previous sections, we have demonstrated that the *audibilization* of the entire FID spectrum, evocative of the harmonious strike of a guitar chord, the ringing of a bell, or the concept of a *molechord* as coined in earlier discussions [4] serves not only as an intriguing method for interpreting complex mixtures but also offers valuable applications in pedagogical contexts, instrument calibration, and beyond. This approach grants a symphonic understanding of the chemical composition, capturing all frequency ranges simultaneously. However, despite its wide-ranging merits, the method presents inherent challenges. Audibly discerning numerous frequencies all at once can be overwhelming, and human capacities for such tasks vary greatly. This raises the question: can we enhance this experience by isolating spectral regions?

To address this, we propose the following procedure: rather than audibly reproducing the entire FID, we focus on “playing” specific regions of a spectrum. In this way, chemists can identify the presence of signals in distinct zones, such as aliphatic, olefinic, or aromatic, purely based on sound. With adequate training, this technique offers a promising avenue for basic structural elucidation, especially as a tool for the visually impaired.

Nevertheless, executing this idea is not without challenges. At first glance, one might think of selecting specific regions, like multiplets, and converting each into its time-domain representation by an inverse Fourier transform, subsequently termed *subFIDs*. These *subFIDs* would then be woven together into a single audio sequence. However, the selection of isolated multiplets or specific spectral regions can introduce discontinuities, demanding the use of window functions to taper off the edges and mitigate potential artifacts. An alternative method might involve using bandpass filtering, where the passband is tailored to the regions of interest. Although this approach might produce a smoother *subFID*, it brings its own set of complexities, including filter design and the potential for phase distortions. Moreover, the process of inverse Fourier transformation, particularly for disjointed spectral regions, can result in artifacts. When dealing with particularly narrow regions, advanced techniques become imperative to achieve a seamless representation.

These complexities underscore the need for a refined approach. Our strategy to circumnavigate these challenges involves deconvolving the spectrum to generate a list of peaks, each characterized by its frequency, intensity, and linewidth. We can employ different algorithms available in Mnova, including GSD or a deep learning-based algorithm [14]. Once this peak list is obtained, synthetic FIDs in the desired regions can be constructed for each multiplet, which are then seamlessly concatenated.

**Figure 3:**
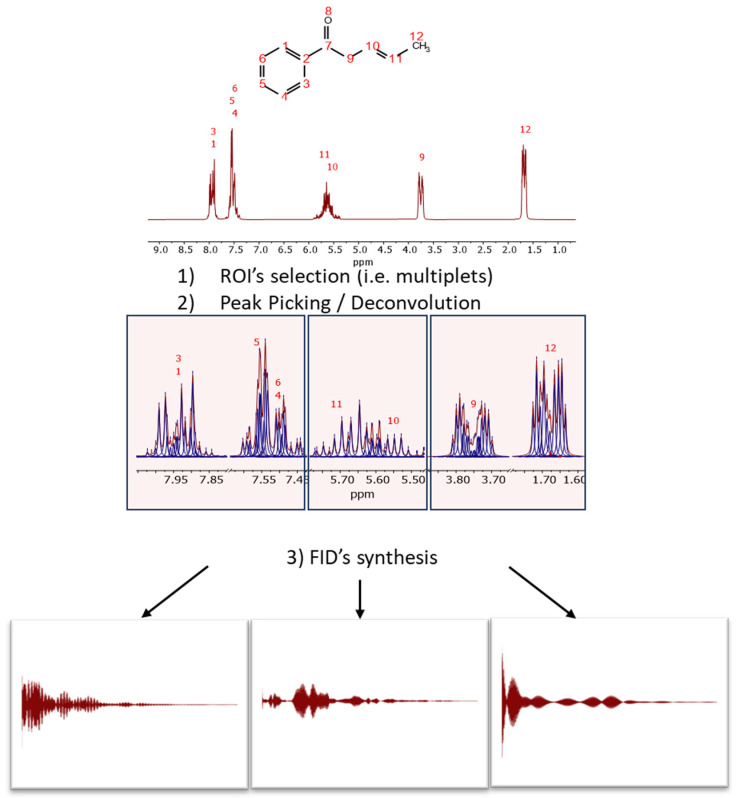
Scheme of audio generation from a spectrum through the synthesis of *subFIDs* in different spectral regions. First, it is necessary to select (manually or automatically) the regions of interest and then perform GSD, so that the resulting peaks will be used to synthesize the different FIDs, assuming a standard model of a pure complex exponential signal.

This process is illustrated in Figure 3 with the predicted spectrum of (3E)-1-phenylpent-3-en-1-one, a molecule specifically designed to exhibit signals in the typical aromatic, olefinic, and aliphatic regions. The spectrum has been synthesized at a spectrometer frequency of 100 MHz (see Figure 3).

The initial step involves selecting the regions of interest (ROIs), either manually or automatically, for instance, using the multiplet analysis feature in Mnova. In each ROI, peak picking is executed. GSD has proven particularly effective for this task due to its rapid and accurate determination of peak parameters.

For each ROI, and from right (high field) to left (low field), an FID is synthesized using the detected peak data, assuming pure exponential decays (e.g., Lorentzian lines). The synthesis formula is:

$$d_{k}=\sum_{j=0}^{L-1}(A_{j}e^{i\Phi_{j}})e^{-k\Delta t/\tau_{j}}e^{2\pi ik\Delta tw_{j}}+\varepsilon_{j}$$

where *L* is the number of resonances (i.e., peaks), and *A_j_*, $w_{j}$, *τ_j_* represent the amplitude, frequency, and relaxation time (*T*_2_, related to linewidth estimated by GSD as $Line\;Width=\frac{1}{\pi T_{2}}$), respectively, for each resonance. In this application, the phase, *ϕ_j_*, is set to zero and the noise, $\varepsilon_{j}$, is omitted).

This method, while still in its exploratory phase, aims to offer a fresh perspective in the audible interpretation of NMR spectra. By emphasizing specific regions of interest, we hope to streamline the listening experience and provide a more inclusive tool tailored to diverse auditory capacities and needs.

## 4. Conclusions

In this work, we present a simple yet practical approach to the acoustic rendering of NMR data, introducing a signal processing formalism that ensures proper alignment of the NMR signals with the audio output. By enabling auditory interpretation of NMR signals, we offer an alternative perspective through which NMR users and educators can experience and interpret NMR data in a complementary way, potentially enhancing understanding and accessibility in certain contexts. Integrating this auditory feature into Mnova, a widely used NMR platform, not only enhances the usability of this auditory experience but also significantly expands its reach within the NMR community. This incorporation paves the way for broader adoption, allowing both seasoned spectroscopists and newcomers to readily access and benefit from the insights provided by the auditory interpretation of NMR data.

The Supplementary Information accompanying this article includes a select dataset of 1H NMR spectra with corresponding WAV sound files, as well as a brief user manual for this new feature in Mnova NMR software.

## Data Availability

The data presented in this study are available in the Supplementary Information file.

## Supplementary Materials

The following supporting information can be downloaded at: https://www.mdpi.com/article/10.3390/molecules29040762/s1, 8 ^1^H NMR spectra as well as 16 sound.

## References

[1] Bloch F., Hansen W.W., Packard M. (1946). The Nuclear Induction Experiment. Phys. Rev..

[2] Purcell E.M., Torrey H.C., Pound R.V. (1946). Resonance Absorption by Nuclear Magnetic Moments in a Solid. Phys. Rev..

[3] Bauer W. (2018). NMR Meets MUSICIANS—How to Get Tones, Sounds and Songs Out of an NMR Spectrometer. YouTube. https://youtu.be/2TTsk7dX4AQ?si=ihRtYo1xqPizXI9e.

[4] Reddy A. (2021). On the use of nuclear magnetic resonance spectroscopy in music composition- principles, practice and possibilities. J. New Music Res..

[5] Morawitz F. Multilayered Narration in Electroacoustic Music Composition Using Nuclear Magnetic Resonance Data Sonification and Acousmatic Storytelling. Proceedings of the 25th International Conference on Auditory Display (ICAD 2019).

[6] Thanos Andreou, Piece “Ipsenol”. https://itaintmagic.riken.jp/research-in-depth/nmr-music-molecules/.

[7] Garrido N., Pitto-Barry A., Soldevila-Barreda J.J., Lupan A., Boyes L.C., Martin W.H.C., Barry N.P.E. (2020). The Sound of Chemistry: Translating Infrared Wavenumbers into Musical Notes. J. Chem. Educ..

[8] Mnova NMR.

[9] Bain A.D., Burton I.W. (1996). Quadrature detection in one or more dimensions. Concepts Magn. Reson..

[10] Sheberstov K.F., Sistaré Guardiola E., Jeannerat D. (2019). Everything you wanted to know about phase and reference frequency in one- and two-dimensional NMR spectroscopy. Magn. Reson. Chem..

[11] Morris G.A. (2017). NMR Data Processing. Encyclopedia of Spectroscopy and Spectrometry.

[12] Audacity. https://www.audacityteam.org/.

[13] Munukutla S., Bertoy A., Rush S., Ramamoorthy A. (2022). Molecular Music: A Modern Accompaniment to NMR Pedagogy. J. Chem. Educ..

[14] Schmid N., Bruderer S., Paruzzo F., Fischetti G., Toscano G., Graf D., Fey M., Henrici A., Ziebart V., Heitmann B. (2023). Deconvolution of 1D NMR spectra: A deep learning-based approach. J. Magn. Reson..

