# Supplementary material for: Molecular Melodies: Unraveling the Hidden Harmonies of NMR Spectroscopy
Source: Molecules. 2024 Feb 7;29(4):762. doi: 10.3390/molecules29040762 (PMC10893351; doi:10.3390/molecules29040762)
Supplement: Supplementary file 1 [file molecules-29-00762-s001.zip › molecules-2809234-supplementary.pdf]

# Molecular Melodies: Unraveling the Hidden Harmonies of NMR Spectroscopy

## Supplementary information

Iria Pérez Varela [1], Gavin Shear [2] and Carlos Cobas [2,*]

[1] Centro de Investigación Mestrelab (CIM), Av. Barcelona 7, 15706 Santiago de Compostela, Spain; iria.perez@mestrelab.com

[2] Mestrelab Research, 15706 Santiago de Compostela, Spain; gavin.shear@mestrelab.com

* Correspondence: carlos@mestrelab.com

## Play FID/NMR in Mnova

The new acoustic rendering feature of NMR signals is available in Mnova NMR 15.0.1 [8] which is currently in the beta stage and subsequent versions. This feature's core functionality has been developed in C++ and integrated into the Mnova software's core, while the user interface is implemented as an Mnova script. The source code for this script is accessible in the 'scripts' directory within the software's installation folder. This new feature can be accessed through the '*Tools/NMR Tools*' menu, as shown in the screen shot below:

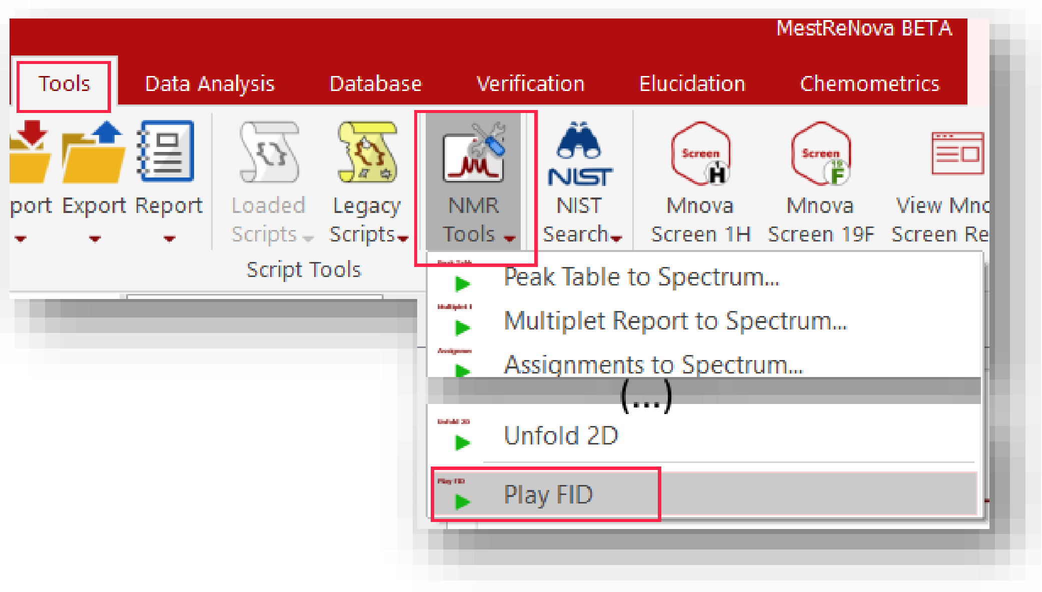


Once this command is issued, the main dialog for this feature will show up as is shown in the figure below:

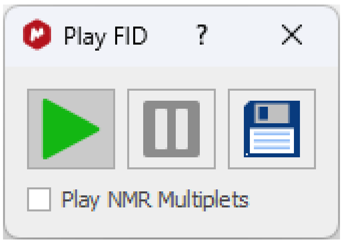


The 'Play', 'Stop', and 'Save' buttons provide intuitive controls, requiring no additional elaboration. It is worth noting that audio files are saved in the WAV format. In contrast, the 'Play NMR Multiplets' checkbox option deserves closer attention. When activated, this novel feature enables acoustic rendering of specific spectral regions, essentially serving as a rudimentary tool for structure elucidation. Prior to its use, it is necessary to define the multiplets in the spectrum. Subsequently, each multiplet is transformed into an FID and played sequentially, from right to left, corresponding to the transition from high to low field.

This feature supports single 1D NMR FID/spectra, as well as stacked FID/spectra from various experiments (reaction monitoring, relaxation, PFG/diffusion experiments, etc). At the moment, 2D NMR spectra are not supported.

1H NMR samples data sets

## Samples 1H NMR spectra

1H NMR spectra of 8 molecules (see table below) have been synthesized using the 1H NMR Prediction capabilities of Mnova. Multiplet analysis has been applied to all these spectra so that the new “Play NMR Multiplets” feature can be tested.

For each 1H NMR spectrum, 3 files are provided:

- ✓ *.mnova: 1H NMR file in Mnova file format
- ✓ *.wav: audio file representing the full FID
- ✓ *_m.wav: audio file generated using the multiplets in each spectrum.

| | | | |
|---|---|---|---|
| 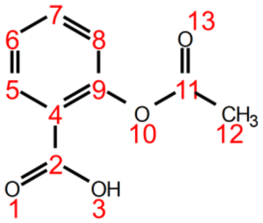 | 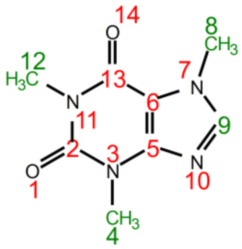 | 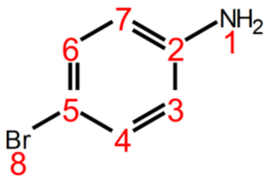 | 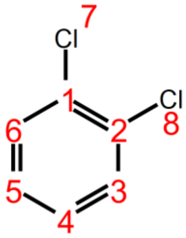 |
| Caffeine | Caffeine | Bromoaniline | ODBC |
| 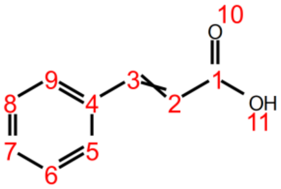 | 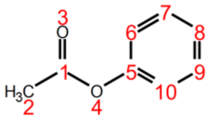 | 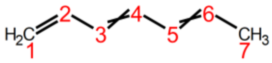 | 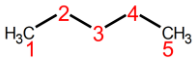 |
| Cinnamic acid | Phenyl acetate | Hepta-1,3,5-triene | Pentane |

## Reference

[8]. Mnova NMR 15.0.1, Mestrelab Research, https://mestrelab.com/software/mnova/nmr/ (accessed on 29 January 2024)
